# Time to definitive care among severely injured farmers compared to other work-related injuries in a Midwestern state

**DOI:** 10.1186/s40621-020-00259-w

**Published:** 2020-07-01

**Authors:** Amanda Swanton, Corinne Peek-Asa, James Torner

**Affiliations:** 1 Department of Surgery, Urology, Dartmouth-Hitchcock Medical Center, Lebanon, NH USA; 2 Occupational and Environmental Health, Injury Prevention Research Center, University of Iowa, 145 N. Riverside Dr, S143 CPHB, Iowa City, IA 52241 USA; 3 Epidemiology, Injury Prevention Research Center, University of Iowa, Iowa City, IA USA

**Keywords:** Acute care, Agricultural injuries, Trauma systems, Trauma registries

## Abstract

**Background:**

Farming is a high risk occupation that predisposes workers to injury, but may also lead to barriers in reaching trauma care. Little is known about emergency and trauma care for patients with farm-related injuries. The purpose of this study was to determine whether severely injured farmers presenting to a statewide trauma system faced delays in reaching definitive care compared to other severely injured workers.

**Methods:**

A population-based observational study was performed using the Iowa State Trauma Registry from 2005 to 2011. The registry was used to identify a multiply imputed sample of severe occupational injuries. Time to definitive care for farm- and non-farm-related injuries was compared using Kaplan-Meier curves and an extended, stratified Cox model censoring at 4 h. An interaction with time was included in the Cox model to generate hazard ratios for each hour after injury.

**Results:**

Seven-hundred forty-eight severe occupational injuries were identified; 21% of these were farm-related. The overall median time to definitive care was nearly an hour longer for farmers compared to other workers (2h46m vs. 1h48m, *p* < 0.05). When adjusted for confounders, farm status remained a significant predictor of delay in reaching definitive care, but only in the first hour after injury (HR = 0.44, 95%CI = 0.24–0.83).

**Conclusions:**

Farm-related injuries accounted for more than 1 of every 5 severe occupational injuries entered into the Iowa trauma system. We found that severely injured farmers had delays in reaching definitive trauma care, even when adjusted for confounding variables such as rurality. This effect was most pronounced in the first hour.

## Background

For decades, agriculture has been recognized as one of the most hazardous occupations in the United States. In 1988, a report released by The National Coalition For Agricultural Safety and Health warned of an “unabating epidemic of traumatic death and injury in American farming” and outlined initiatives to improve the working conditions of agricultural workers (Merchant et al. 1988). However, the burden of injury in agriculture has remained high with an estimated one in 20 farmers experiencing an injury every year (Myers 1997; Myers 1998; Myers 2001; Gerberich et al. 1993). One strategy for improving the outcomes following an injury is rapid access to trauma services that can provide the necessary level of care.

Trauma systems arose from a need to provide care for the numerous injuries that occur among the general population, and evidence suggests that such systems have been beneficial at a population level. The results of a recent meta-analysis of 6 population-based studies indicated that trauma system implementation was associated with an estimated 15% reduction in mortality (Celso et al. 2006). However, despite this improvement in outcomes, the benefits may not have been realized in all populations. For example, while the implementation of the Oregon trauma system resulted in a decreased risk of death on a state-level (adjusted OR = 0.80, CI_95%_ = 0.70–0.91) (Mullins and Mann 1998), the same benefit could not be replicated in a remote rural sample from the same state (Mann et al. 2001).

Most trauma systems were developed in urban areas, and most information evaluating trauma system effects are from urban systems. While farmers are among those served by trauma systems, there is reason to suspect that farmers have many barriers to reaching timely trauma care. Rurality itself has repeatedly been identified as a risk factor for delays in reaching definitive care (Carr et al. 2005; Esposito et al. 1995; Grossman et al. 1997; Rogers et al. 1997; Spaite et al. 1993; Gonzalez et al. 2006; Stripe and Susman 1991), but it is likely that other features of farm work also serve as barriers in the trauma response process (Stueland et al. 1993). Farmers often work unsupervised for long periods of time, and their injuries may be more likely to go undiscovered. Once EMS is notified, providers may face difficulties reaching the injured farmer due an imprecise location description, poor road access, and hazards at the scene (e.g. animals, machinery, and inclement weather). Additionally, previous work has shown that farmers may be less likely to arrive by ambulance than other patients presenting to rural hospitals (Young et al. 2003), which not only suggests difficulty or reluctance in accessing EMS services, but also indicates that farmers may be missing the benefit of formal triage by an EMS provider.

Despite the high incidence of agricultural injury and the potential barriers to the subsequent trauma response, no studies have examined how long it takes farmers to reach definitive trauma care, care which provides all the patient’s requirements for specialized treatment, in a modern trauma system. This study used injury surveillance data to perform a retrospective cohort study among those experiencing occupational injuries in the state of Iowa in order to determine whether time to definitive care differs for injured farmers compared to other workers. Such information will not only be valuable for system evaluation at a state level, but will also be informative for other states with trauma systems that serve sizeable agricultural communities.

## Materials and methods

The state of Iowa has an inclusive trauma system in which all hospitals within the state are categorized based on the level of care (Level I-IV) available at a given facility in accordance with the designations outlined by the American College of Surgeons (American College of Surgeons 2014). The majority of hospitals in the state are small community hospitals (Level IV) that provide definitive care for relatively minor injuries, while a minority of hospitals serve as resource (Level I) or regional (Level II) trauma centers capable of providing definitive care for the most severe injuries. Emergency Medical Systems (EMS) providers statewide use standard triage protocols to evaluate the severity of a given injury and determine the appropriate level of care (Iowa Department of Public Health 2015).

A key component of the Iowa trauma system is the Iowa State Trauma Registry (ISTR), a state-mandated active injury surveillance registry maintained through the Iowa Department of Public Health (Iowa Trauma Patient Data Dictionary 2005). Trauma centers in the State of Iowa report demographic, prehospital, and injury information for patients that meet trauma criteria that are evaluated or treated at that trauma center. Patients are eligible for the registry if they are evaluated or treated at an Iowa hospital. Patients are also eligible if the trauma team, which may include physicians, nurses, and support staff, is mobilized prior (i.e. during ambulance transport) or upon the patient’s arrival at the hospital. Though an individual may receive care from more than one provider or facility, the final injury report is filed by the definitive care hospital using an electronic reporting tool.

Eligible patients were adults (≥ 16 years of age) that sustained and sought treatment for a severe (injury severity score ≥ 16) work-related injury in the state of Iowa between 2005 and 2011. Age 16 and over was used because the state of Iowa issues work permits at the age of 16. Although younger youth are involved in many work activities, especially on the farm, comparisons of younger ages would have small numbers and different types of occupational distributions. The sampling for this study occurred in multi-step process. First, adults with work-related injuries were identified in the ISTR as part of a larger dataset including injuries of all severities. As defined by the ISTR, work-related is defined as occurring while an individual is being compensated, while they are at or traveling to a their place of work, and while performing a job-related activity (Iowa Trauma Patient Data Dictionary 2005). Patients who were injured or received care outside of the state of Iowa were excluded.

Within the ISTR is a designation of whether or not the injury was farm-related. Eligible severe, work-related injuries were categorized into two exposure groups: farm-related (farm) and not-farm-related (non-farm), and this served as the primary exposure variable. The main outcome in this study was time to definitive care which was calculated as the time interval between the injury event and arrival at the definitive care hospital. Additional covariates including patient, injury, and scene characteristics were also ascertained from the registry. Rurality was determined using The Rural Urban Commuting Area Codes (RUCA), maintained by the US Department of Agriculture, which designate rurality based on population density, urbanization, and daily commuting (RUCA Zip Code Data 2004). Using standard RUCA coding guidelines, a 4-level categorical measure of rurality was derived from the zip code of the injury scene. Urban was defined as zip codes in urbanized areas (pop. ≥50,000) or those with at least 30% commuting flow to an urbanized area. Large town, small town, and rural zip codes were those in large urban clusters (pop. 10,000-49,999), small urban clusters (pop. 2500-9999), and outside an urban cluster (pop. < 2500), respectively, that had less than 30% commuting flow to an urbanized area.

Once the sample was identified, multiple imputation by chained equations was performed using a program called SRCware (University of Michigan 2014) to complete any observations with missing predictor information. Benefits of this imputation method include the ability to estimate values for categorical variables and to calculate estimates for patients with missing values in more than one variable (10.1% of the patients identified). Of the variables, rurality was the most frequently missing (16.4%); all other variables had < 15% missing. We developed 10 imputed datasets, which were then sampled for only severe injuries. Because the severity variable itself was imputed, the number of patients in each imputed sample varied (range = 729–776).

### Analysis

Survival modeling, using both Kaplan-Meier analysis and Cox proportional hazard modeling, was used to evaluate time to definitive care using right censoring at 4 h. Although a majority of acute care studies use 60 min as the benchmark for definitive care (Lerner and Moscati 2001), this time period is often infeasible in rural areas given transport distance to a healthcare facility. The State Trauma System uses 4 h as the benchmark indicator, and we have adapted this system indicator for our analysis. Individuals with missing time to definitive care data (12%) were retained in the analysis, but were censored at 4 h.

Kaplan-Meier curves were constructed for time to definitive care among farmers and non-farmers; the differences between these curves were tested using the log-rank test. *P*-values are reported as the most conservative estimate among the ten imputed sets.

A Cox proportional hazard model was attempted to adjust for potential confounders. Covariates were added into the stratified model based on both a priori hypotheses and empirical analysis of the Akaike information criterion (AIC). However, several variables, including rurality and farm status, were found to violate the proportionality assumption. To address this, the model was modified to include a time-dependent interaction with farm and to stratify on rurality (stratified, extended Cox model). The final model used four heaviside functions to evaluate the farm vs. non-farm relationship for each of the one-hour blocks within the 4-h observation period. The intervals were defined as follows, where t = time in hours:

$$ {\displaystyle \begin{array}{c}{g}_1(t)=\left\{\begin{array}{c}1\  if\ t<1\\ {}0\  if\ t\ge 1\end{array}\right.\\ {}{g}_2(t)=\left\{\begin{array}{c}1\  if\ t<2\  and\ t\ge 1\\ {}0\  if\ t<1\  or\ t\ge 2\end{array}\right.\\ {}{g}_3(t)=\left\{\begin{array}{c}1\  if\ t<3\  and\ t\ge 2\\ {}0\  if\ t<2\  or\ t\ge 3\end{array}\right.\\ {}{g}_2(t)=\left\{\begin{array}{c}1\  if\ t\ge 4\\ {}0\  if\ t<4\end{array}\right.\end{array}} $$

As a result, a separate hazard ratio was generated for each hour after injury.

All analyses were conducted in SAS v.9.4 (SAS Institute Inc 2011) using PROC LIFETEST for the Kaplan-Meier analysis, PROC PHREG for the Cox modeling, and PROC MIANALYZE for pooling results from imputed sets. Reported estimates represent pooled results unless otherwise stated.

## Results

From 2005 to 2011, an average of 748 severely injured workers were identified in the ISTR and included in this analysis; 21% of these severe injuries were farm-related. The characteristics of the severe farm- and non-farm-related injuries are shown in (Table 1). Severely injured farmers tended to be older and injured in more rural environments. Farmers were also less likely to pay via worker’s compensation and more likely to pay by private insurance or Medicaid/Medicare. Farmers and non-farmers appeared comparable in sex distribution, frequency of night and weekend injuries, and injury type. With regards to nature of injuries, pelvic fractures were significantly more frequent among farmers, while long bone fractures were significantly less frequent. Crush injuries and amputations were relatively uncommon and occurred in less than 5% of cases in both groups. Additionally, the majority of severe injuries were transported by Emergency Medical Services (EMS), including similar proportions of farmers (79%) and non-farmers (86%).

**Table 1:** Characteristics of severely injured occupational injuries, by farm status. Percentages are reported as an average across the 10 imputed sets

	Farm Work-Related Injury (*n* = 158)^a^	Non-Farm Work-Related Injury (*n* = 590)^a^	
	%	%	p
**Age**			< 0.0001
16–24	6.3	10.1	
25–44	20.4	34.8	
45–64	41.2	46.6	
> =65	32.1	8.5	
**Sex**			0.3078
Male	94.6	92.0	
Female	5.4	8	
**Primary Payer**			< 0.0001
Insurance	21.7	9.0	
Medicaid/Medicare	12.7	3.8	
Worker’s Comp	16.4	47.6	
Other/Unknown	49.2	39.7	
**Time**			0.4286
Night (6:00 pm – 5:59 am)	10.0	14.2	
Day (6:00 am - 5:59 pm)	90.0	85.8	
**Weekend**			0.1328
Yes	19.9	14.6	
No	80.1	85.4	
**Rurality**			< 0.0001
Urban	22.9	56.7	
Large Town	11.7	12.7	
Small Town	21.6	15.9	
Isolated Rural (non-town)	43.7	14.6	
**Injury Type**			0.7133
Blunt	95.1	94.8	
Penetrating	0.6	1.4	
urn	4.3	3.8	
**Nature of Injury**			
Pelvic Fracture	18.9	10.6	0.0073
Amputation	0.7	0.5	0.7905^b^
Long Bone Fracture	7.5	21.3	< 0.0001
Spinal Injury	38.9	34.7	0.3798
Crush Injury	3.4	2.4	0.5150
Brain/Skull Injury	49.4	57.9	0.0769
Chest Injury	7.4	7.5	0.8079
**EMS Use**			0.1315
User	79.0	86.0	
Non-user	21.0	14.0	

^a^Reported as an average. Numbers for individual imputed sets vary

^b^Estimated from Chi-Square test despite cell counts < 5

**Fig. 1:**
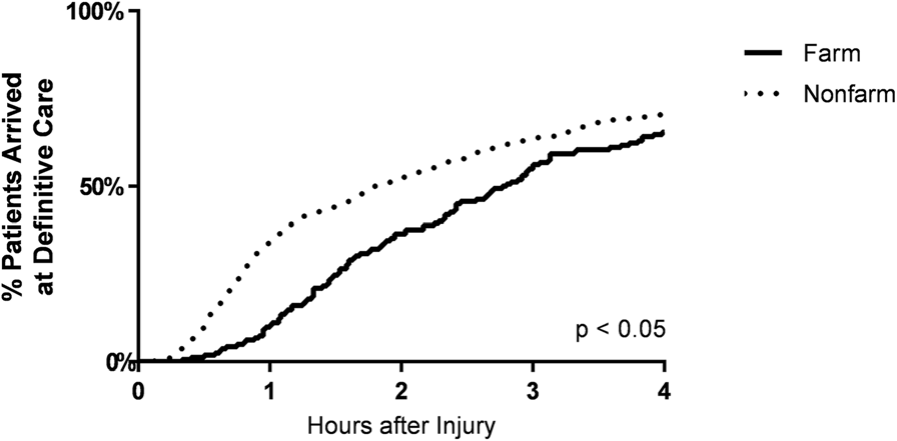
Kaplan-Meier curves showing the probability of having reached definitive care (censored at 4 h) among severely injured farmers and non-farmers in the first imputed set. *P*-values were obtained using the log-rank test and are reported as the most conservative (i.e. least significant) value among all imputed sets

Kaplan-Meier curves were constructed to show the probability of reaching definitive care for farmers and non-farmers and indicated that farmers were significantly (*p* < 0.05) less likely to reach care in a given time period (Fig. 1). The median time to definitive care was approximately 1 h longer for farmers (2h46m, range: 2h40m – 2h54m) compared to non-farmers (1h48m, range: 1h48m – 1h58m). However, these curves were unadjusted for other covariates, such as rurality.

Table 2 shows results from the Cox proportional hazard model with the hazard ratio calculated for each hour. In the first hour after injury, time to definitive care was longer for farmers than other work-related injuries (HR = 0.44, 95%CI = 0.24–0.83). In contrast, the estimates for hours 2 through 4 suggest the opposite effect; however, this fails to meet statistical significance. Taken together, our findings suggest that farmers may face early barriers to reaching care; however, the effects of these barriers do not appear to persist. When stratified by EMS use, the delay in the first hour was significant among EMS users (HR = 0.42, 95%CI = 0.22–0.80), but did not reach significance among EMS non-users. While this could indicate that this delay only exists among EMS users, this could also be a result of the small sample size (avg. *n* = 116) in the EMS non-user group.

**Table 2:** Adjusted hazard ratios from Cox proportional hazard model for predictors of time to definitive care among severe occupational injuries. Note: HR < 1 indicates a longer time to definitive care*

	All Severe Injuries		Subset: EMS Non-users		Subset: EMS Users	
	adjHR	95% CI	adjHR	95% CI	adjHR	95% CI
**Farm vs. Non-farm**						
One hour and under	*0.45*	*(0.24,0.83)*	0.71	(0.16,3.09)	*0.42*	*(0.22,0.80)*
61 to 120 min	1.33	(0.89,1.98)	0.81	(0.13,5.07)	1.46	(0.95,2.23)
121 to 180 min	1.28	(0.79,2.08)	1.96	(0.42,9.12)	1.15	(0.65,2.05)
181 to 240 min	1.40	(0.77,2.54)	0.63	(0.11,3.65)	1.73	(0.84,3.59)
**Injury Type (Yes vs. No)**						
Pelvic Fracture	1.05	(0.77,1.42)	1.67	(0.30,9.43)	1.01	(0.74,1.38)
Long Bone Fracture	*1.32*	*(1.02,1.71)*	*3.59*	*(1.14,11.29)*	1.20	(0.92,1.56)
Spine Injury	1.05	(0.86,1.29)	*1.99*	*(1.01,3.92)*	0.96	(0.78,1.18)
Brain Injury	0.94	(0.77,1.15)	0.92	(0.46,1.81)	0.96	(0.78,1.19)
Chest Injury	0.95	(0.67,1.34)	0.25	(0.03,2.35)	1.03	(0.72,1.47)
**Additional Covariates**						
*Payer*						
Insurance	ref		Ref		ref	
Medicaid/Medicare	0.94	(0.56,1.59)	0.59	(0.12,2.82)	0.93	(0.53,1.64)
Worker’s Comp	0.87	(0.63,1.20)	0.67	(0.23,1.93)	0.88	(0.63,1.24)
Other/Unknown	*0.58*	*(0.42,0.80)*	*0.35*	*(0.13,0.91)*	*0.61*	*(0.43,0.85)*
*Age*						
16–24	*0.70*	*(0.50,0.98)*	0.74	(0.19,2.96)	*0.67*	*(0.47,0.97)*
25–44	ref		Ref		ref	
45–64	*0.80*	*(0.65,0.99)*	0.74	(0.39,1.40)	0.81	(0.64,1.02)
> =65	*0.64*	*(0.45,0.90)*	0.84	(0.32,2.24)	*0.62*	*(0.41,0.92)*

*: Italicized text indicates effect estimates and 95% confidence intervals that are statistically significant. All variables were included in each model

The adjusted hazard ratios for other variables included in the model are shown in Table 2. While the types of injury related to trauma triage were included in the model, only the presence of a long bone fracture was a significant predictor of increased time to definitive care (HR = 1.32, 95%CI = 1.02–1.71). The increased hazard of reaching care (decreased delay) for long bone fracture patients was even stronger among EMS non-users (HR = 3.59, 95%CI = 1.14–11.29), though the wide confidence intervals are again noted; long bone fracture was not a significant predictor among EMS users. Though not significant among all severe injuries or the EMS subset, spine injuries were also associated with decreased delay in EMS non-users (HR = 1.99, 95%CI = 1.01–3.92). Pelvic fractures, brain injuries, and chest injuries all had hazard ratios close to 1 in the all severe injuries model and were not significant predictors of time to definitive care in this model or in either EMS subset.

Primary payer and age also entered the model as informative predictors of time to definitive care. Compared to privately insured patients, worker’s compensation and Medicaid/Medicare patients were not significantly different with regard to reaching care; however, self-paying or having an unknown insurance status was associated with a delay (HR = 0.58, 95%CI = 0.42–0.80); this was consistent among both EMS subsets. Certain age groups were also associated with decreased hazard of reaching care in the all severe injuries model, particularly younger and older workers. For younger workers aged 16–24 and older workers aged 65 and older, the hazard for reaching care was approximately two-thirds the hazard rate of adults aged 25–44. This relationship was less pronounced, but still significant for adults aged 45–64 who had 80% the hazard of reaching care compared to those 25–44. The effect estimates for these age groups suggested delay in both EMS subset, but only reached statistical significance for younger and older workers who used EMS.

## Discussion

Farm-related injuries comprised more than 1 of every 5 occupational injuries encountered by the Iowa trauma system. Notably, nearly a third of the farm-related injuries were in older workers, which have previously been identified as a high risk group due to the increased morbidity and mortality associated with injury (Myers et al. 2009).

Due to the nature of agricultural work, we hypothesized that severely injured farmers were more likely to face delays than non-farmers, and according to both Kaplan-Meier and Cox analysis among all severely injured workers, there was indeed evidence of significant delay among farmers compared to non-farmers (Fig. 1, Table 2). Since the Cox analysis adjusted for rurality, this delay cannot be attributed to distance from a trauma center. The addition of a time-dependent interaction in the Cox analysis also showed that this delay was restricted to the early portion of the prehospital period, specifically to the first hour after injury.

To our knowledge this is the first study to identify that a delay in reaching trauma care exists specifically for farmers. While several previous studies have identified delays among rural populations (Carr et al. 2005; Esposito et al. 1995; Grossman et al. 1997; Rogers et al. 1997; Spaite et al. 1993; Gonzalez et al. 2006; Stripe and Susman 1991), none have attempted to separate the effects of rurality from those of farming. Rurality contributes to increased prehospital time based on distance to definitive care, but this research indicates there are likely additional factors that affect farmers; these factors may include working alone, being injured in locations difficult to access, and the presence of hazards (i.e. animals, machinery, natural elements) at the scene (Gerberich et al. 1993; Mullins and Mann 1998; Mann et al. 2001). While our study suggests that farmer-specific factors are contributory, our study is not able to specify which factors are most important. Additional analyses are needed to explicitly identify influential factors and ultimately lead to interventions that reduce delay in trauma care for farmers. Further qualitative work examining the barriers to injury response in detailed injury narratives, which are less likely to be available from registry and/or hospital data, is also needed.

Delays experienced by farmers were confined to the first hour after injury; farmers had more than a 50% reduction in hazard of reaching care during this period. The subset analysis of Emergency Medical Services (EMS) users and non-users (Table 2) showed that EMS users mirrored the results of the entire sample, while the delays among non-users were not significant. This non-significant finding could be due to the small sample size. However, it is also possible that the prehospital experiences of EMS users and non-users truly differ. In either case, the fact that the sample of severe injuries was predominately EMS users means that the findings of the overall model are more reflective of the experience of EMS users.

Many factors could contribute to the delays found in the first hour after injury. First, this finding may indicate that the early stages in process of reaching care are delayed (e.g. identifying that an injury has occurred) in farmers, while later stages (e.g. driving to the trauma center) are no different. The comparison group, which included non-farm work-related severe injuries, is important for the interpretation of these findings. Since our analysis of time to definitive care for severely injured workers (Table 2) compares farmers to all other non-farm Iowan workers, there could be a subset of non-farm workers (e.g. hospital workers) that reach care quickly relative to both the farmers as well as the other controls and could account for the early increase in hazard of reaching care among controls. Since the ISTR does not contain occupational designations for the non-farm workers this is challenging to assess in the current dataset; such a variable would aid future studies of occupational injury using the ISTR. Though the root cause of this delay is unclear, our findings do warrant further investigation into the ability of farmers to access trauma services following a severe injury.

Several other variables were found be predictive of time to definitive care for all severe injuries. Of the injury types, only long bone fracture was significant in the model for all severe injuries and was associated with decreased delay in reaching definitive care. While other injury types may necessitate a surgical specialist, previous studies have shown that rural general surgeons routinely perform orthopedic procedures (Breon et al. 2003; Landercasper et al. 1997), perhaps allowing some patients with severe long bone fractures to be handled locally. Age was also a significant predictor with the youngest and oldest workers facing greater delays. Pediatric specialty services in Iowa are concentrated in two urban regions, which may lead to long travel times in this demographic. Older adults are more likely to be under-triaged (Chan et al. 2006; Rogers et al. 2012; Haas et al. 2010; Chang et al. 2008) to lower level trauma centers, which may lead to delayed transfer and increase the time to definitive care. Older individuals may also be more medically fragile necessitating stabilization at a local facility before proceeding to definitive care. Lastly, having an other/unknown insurance payer predicted increased delay. This likely represents a data collection inefficiency for transferred patients in which insurance information is collected by the first hospital, but is not relayed to the definitive care hospital, which is ultimately responsible for reporting to the registry.

### Limitations

Several limitations need to be considered. First, the ISTR includes only patients who sought care at a hospital or for whom a trauma alert was initiated. This does not include those who chose not to seek medical attention or who sought treatment at an outpatient clinic, leading to an under-sampling of the true statewide injury burden. We limited the influence of non-treatment by examining only severe injuries, which are likely to require acute medical care. However, this sample may not be generalizable to all injured individuals. One subset of severe injuries includes those who die at the scene, and these are not captured in the ISTR. Given that approximately 1 in 1000 occupational injuries is fatal, we estimate that very few individuals were missed due to death prior to EMS arrival (*n* < 6) and suspect that their exclusion would not have had a major impact on our analyses.

Data coding quality and missing data are limitations with any hospital data. Data is input by trauma nurse coordinator following a well defined codebook, and hospitals undergo annual data quality checks. However, the extent of data miscoding for the occupational and farm variables is unknown. We anticipate few false positives (if the injury is coded as farm-related it is most likely farm-related), but false negatives may be more common. For missing predictors, we made the assumption that data are missing at random and performed multiple imputation prior to analysis. This process allowed us to retain all observations ascertained from the ISTR for subsequent analyses, which reduces but doesn’t eliminate the risk of biased results. The outcome variable, time to definitive care, was also missing in approximately 12% of our sample. In our analysis, those with missing outcomes were retained, but censored at 4 h (i.e. treated as lost to follow-up). A sensitivity analysis using an alternate approach of excluding those with missing data from the survival model yielded nearly identical results.

Lastly, the ISTR also does not collect data for several variables that may have been useful in this analysis. First, GPS coordinates of the injury scene were not available, so rurality was defined based on zip code level data; a more precise definition of rurality based on distances may have allowed for a more complete adjustment for rurality. Second, no information is collected regarding the industry of non-farm workers, which limits our ability to compare farm workers with workers in other specific injuries and should be considered when generalizing these results to other states. Lastly, while the ISTR does contain information about fatality, no measures of disability are collected. Therefore, though our analyses have identified delays, it is difficult to determine what clinical impact these delays have on patient outcomes. Further studies are needed to determine what level of delay equates with measurable patient harm.

## Conclusion

To our knowledge, this is the first study examining access to trauma services for severely injured farmers. Consistent with our hypothesis, farmers were shown to face delays in reaching definitive trauma care relative to other workers even after adjustments for potential confounders, such as rurality. In particular, these effects were confined to the first hour after injury and may suggest that events in the initial stages of the trauma response may be contributing to delay. Further work is needed to identify not only the barriers faced by farmers, but modifiable targets within the trauma system that could lead to a more efficient trauma response to agricultural injury.

## Data Availability

The Iowa Trauma Registry Data are available from the Iowa Department of Public Health through a Data Use Agreement with the Research and Ethics Review Committee: https://idph.iowa.gov/PublicHealthData/research-requests.
